# Profiling of transcriptional regulators associated with starch biosynthesis in sorghum (*Sorghum bicolor* L.)

**DOI:** 10.3389/fpls.2022.999747

**Published:** 2022-08-30

**Authors:** Qianlin Xiao, Tianhui Huang, Wan Cao, Kuang Ma, Tingting Liu, Fangyu Xing, Qiannan Ma, Hong Duan, Min Ling, Xianlin Ni, Zhizhai Liu

**Affiliations:** ^1^College of Agronomy and Biotechnology, Southwest University, Chongqing, China; ^2^Rice and Sorghum Research Institute, Sichuan Academy of Agricultural Sciences, Deyang, China; ^3^Sichuan Sub Center, National Sorghum Improvement Center, Luzhou, China

**Keywords:** sorghum (*Sorghum bicolor* L.), starch biosynthesis, transcription factor, *cis*-element, transcriptional regulation

## Abstract

Starch presents as the major component of grain endosperm of sorghum (*Sorghum bicolor* L.) and other cereals, serving as the main energy supplier for both plants and animals, as well as important industrial raw materials of human beings, and was intensively concerned world widely. However, few documents focused on the pathway and transcriptional regulations of starch biosynthesis in sorghum. Here we presented the RNA-sequencing profiles of 20 sorghum tissues at different developmental stages to dissect key genes associated with sorghum starch biosynthesis and potential transcriptional regulations. A total of 1,708 highly expressed genes were detected, namely, 416 in grains, 736 in inflorescence, 73 in the stalk, 215 in the root, and 268 genes in the leaf. Besides, 27 genes encoded key enzymes associated with starch biosynthesis in sorghum were identified, namely, six for ADP-glucose pyrophosphorylase (AGPase), 10 for starch synthases (SSs), four for both starch-branching enzymes (SBE) and starch-debranching enzymes (DBEs), two for starch phosphorylases (SPs), and one for Brittle-1 (BT1). In addition, 65 transcription factors (TFs) that are highly expressed in endosperm were detected to co-express with 16 out of 27 genes, and 90 *cis*-elements were possessed by all 27 identified genes. Four NAC TFs were cloned, and the further assay results showed that three of them could *in vitro* bind to the CACGCAA motif within the promoters of *SbBt1* and *SbGBSSI*, two key genes associated with starch biosynthesis in sorghum, functioning in similar ways that reported in other cereals. These results confirmed that sorghum starch biosynthesis might share the same or similar transcriptional regulations documented in other cereals, and provided informative references for further regulatory mechanism dissection of TFs involved in starch biosynthesis in sorghum.

## Introduction

Starch is the most important storage format of carbohydrate in plants, and is widely used in human food, animal feed, industrial raw materials, and also as an energy supplier for plant growth and development (James et al., 2003). Starch mainly exists in cells in the form of starch granules, which are mainly composed of two major components, i.e., amylose and amylopectin (Buléon et al., 1998). Amylose is essentially a linear polymer consisting of thousands of glucose residues joined *via* α-1,4 linkages, whereas amylopectin is a larger polymer regularly that contains linear chains of various lengths and a highly branched glucan joined *via* α-1,6 linkages (Myers et al., 2000). Starch is the main component of grain in many cereal crops, and its biosynthesis and accumulation also affect the final performance of yield and quality of these cereals (Bahaji et al., 2014).

Starch biosynthesis occurs in the plastid of higher plants depending on four enzymes, namely, ADP-glucose pyrophosphorylase (AGPase), starch synthase (SS), starch-branching enzyme (SBE), and starch-debranching enzyme (DBE) (James et al., 2003; Hennen-Bierwagen et al., 2009). AGPase catalyzes Glu-1-P combined with ATP to form ADP-glucose (ADPG), a key step that provides a direct substrate for starch biosynthesis (Martin and Smith, 1995; Bahaji et al., 2014). There are two types of SS in plants responsible for the biosynthesis of different types of starch, i.e., granule-bound starch synthase (GBSS) and soluble starch synthesis (SSS). GBSS is the key enzyme for the synthesis of amylose (Sano, 1984; Delrue et al., 1992; Nakamura et al., 1995), and SSS catalyzes the elongation of the chain by adding glucose from ADPG to the non-reducing end of the acceptor chains, playing important role in the synthesis of amylopectin with different chain lengths (Ball and Morell, 2003; Keeling and Myers, 2010). SBE catalyzes the formation of the starch branch by breaking α-1,4 bonds and re-attaching the reducing ends of the glucan chains by α-1,6 bonds to another (or the same) glucan chain (Blauth et al., 2001; Zeeman et al., 2010). Two types of DBEs, isoamylase (ISA) and pullulanase (PUL) (Kubo et al., 1999; James et al., 2003) participate in amylopectin synthesis *via* hydrolyzing the wrong branch linkages and it is also involved in the degradation of starch and formation of starch granules (Myers et al., 2000; Burton et al., 2002; Zeeman et al., 2007; Lü et al., 2008).

In addition to these four major enzymes, some other proteins or enzymes also function in the biosynthesis or accumulation of starches in plants. Brittle-1 (BT1) is responsible for the transmembrane transportation of ADPG (Shannon et al., 1996, 1998), and it was documented that mutant of *bt1* tended to result in the accumulation of ADPG in the cytoplasm, the decrease of starch content, and the limitation of grain development (Shannon et al., 1996, 1998; Cakir et al., 2016). Starch phosphorylase (SP) has been reported to participate in starch biosynthesis through interaction with other functional enzymes (Tickle et al., 2009; Nakamura et al., 2012). It was documented that SP, also known as α-glucan phosphorylase, could function together with SBE to accelerate the synthesis of maltodextrin to initiate the starch biosynthesis in rice endosperm (Nakamura et al., 2012). Besides, the CRISPR/Cas9-based edited mutants of α-glucan phosphorylase-related gene, i.e., *OsPho1*, in rice exhibited significantly increased gene expression level, companioned with the increasing expressions of *AGPS*- and *ISA*-related genes, and *BT1*, as well (Liu et al., 2021).

Starch biosynthesis in plants depends not only on functional enzymes but also on diverse levels of regulation (Jeon et al., 2010; Zeeman et al., 2010; Bahaji et al., 2014), among which transcriptional regulation is an important way (Fu and Xue, 2010; Wang et al., 2013, 2020). In rice, *OsYB1* (Bello et al., 2019), *OsbZIP58* (Wang et al., 2013), *OsRSR1* (Fu and Xue, 2010), *OsNAC20*, and *OsNAC26* (Wang et al., 2020) were reported to mediate the starch biosynthesis through transcriptional regulating of starch biosynthesis-related genes. In maize kernels, numerous transcription factors (TFs), namely, *ZmbZIP91* (Chen et al., 2016), *ZmEREB156* (Huang et al., 2016), *ZmMYB14* (Xiao et al., 2017), *ZmDof3* (Qi et al., 2016), *O2*, and *PBF* (Zhang et al., 2016), *ZmNAC34*/*126*/*128*/*130* (Xiao et al., 2021) also play important roles in starch biosynthesis. Furthermore, *TaNAC019* (Gao et al., 2021) and *TubZIP28* (Song et al., 2020) are important regulators of starch biosynthesis in wheat. *HvSUSIBA2* is a TF involved in starch biosynthesis in barley (Sun et al., 2003), and this TF can also affect the yield performance of rice varieties after overexpression in rice (Su et al., 2015). All these TFs are involved in transcriptional regulations of starch biosynthesis-related genes by binding the specific *cis*-elements. For example, the sugar-responsive elements in the *iso1* promoter are the direct binding site of HvSUSIBA2 (Sun et al., 2003), ACTCAT element is the direct binding site of ZmbZIP91 (Chen et al., 2016), and ZmNAC126 usually binds to tandem repeats of CACG (Xiao et al., 2021), while AAAG (P box) and ACGT (O2-box) are the binding sites of O2 and PBF in *ZmSSIII* promoter (Zhang et al., 2016), and ACGCAA is the binding element of ZmNAC128/130 (Zhang et al., 2019). Therefore, TFs and *cis*-elements directly constitute the basis of transcriptional regulation of starch biosynthesis in different crops, while the transcriptional regulation of starch biosynthesis was rarely reported in sorghum.

Here we dissected the expression profiles of 20 samples belonging to eight different tissues in sorghum at different growth stages, and all starch biosynthesis-related genes were detailed and documented, including their colinear dissection with *Arabidopsis* and maize, *in vitro* binding, and co-expression assays with selected TFs. The summary results of this study uncovered a genome-wide view of starch biosynthesis-related genes and the potential transcriptional regulation profile in sorghum, and provided informative references for further mechanism exploring, especially the dissection of the regulatory landscape of starch biosynthesis in sorghum grains, as well.

## Materials and methods

### Plant materials and sample preparation

Sorghum cultivar of BTx623, kindly provided by Rice and Sorghum Research Institute, Sichuan Academy of Agricultural Sciences, China, was grown on the farm of Southwest University with standard irrigation and fertilization. For RNA-sequencing (RNA-seq) and gene expression analysis, the root, stem, leaf, leaf vein, leaf sheath, seeds, and endosperm were obtained at seedling, jointing, heading, and maturity stage. All the tissues/samples were immediately frozen in liquid nitrogen and then stored at −80°C. Three biological replicates were taken for each sample.

### Transcriptome analysis

All prepared sorghum tissues were collected and used for RNA isolation with TRIzol regent (Invitrogen, Carlsbad, CA, United States) according to the manufacturer’s instructions. NEBNext^®^ UltraTM RNA Library Prep Kit for Illumina^®^ (NEB, United States) was used to generate libraries, and purified libraries were sequenced on an Illumina NovaSeq platform (Novogene, Beijing, China). Clean reads were obtained by removing reads containing adapter, reads containing ploy-N and low-quality reads from raw data, and aligned to the reference genome using Hisat2 v2.0.5. FeatureCounts v1.5.0-p3 was used to count the reads numbers mapped to each gene. Fragments Per Kilobase of transcript sequence per Millions (FPKM) were used for estimating gene expression levels. Differential expression analysis of two conditions was performed using the edgeR R package (3.18.1). The *P*-values were adjusted using the Benjamini and Hochberg method and a corrected *P*-value of 0.05 and absolute foldchange of 2 were set as the threshold for significantly differential expression.

### Sequence attained, annotation, nomenclature, and phylogenetic analysis

To attain the genes of the starch biosynthesis pathway in the sorghum genome, we collected the starch biosynthesis-related genes reported in *Arabidopsis thaliana*, and maize (*Zea mays* L.) (Supplementary Table 1). BLASTP was further performed to search the sorghum genome through the databases of Gramene^1^ and NCBI^2^ with maize starch biosynthesis-related genes as queries. Clustalw1.83 and SMART^3^ were used for the protein sequence alignments and confirmation of the conserved domains, respectively (Letunic and Bork, 2018). MEME^4^ was used for the conserved motifs investigation (Bailey et al., 2015). The minimum and maximum motif width and the number of different motifs were specified as 6, 50, and 10, respectively. The molecular weight (Mw) and isoelectric point (pI) were calculated *via* Swiss-Prot/TrEMBL^5^.

Evolutionary trees of starch biosynthesis-related genes were constructed *via* MEGA 5.10, and the neighbor-joining (NJ) method was used with a p-distance model, and the bootstrap replicates were set to 1,000 (Tamura et al., 2011). According to the genome annotation of the starch biosynthesis-related genes, the chromosome locations, duplication, and gene collinearity were analyzed by local BLASTP and MCScanX. Circos was used to display the results (Krzywinski et al., 2009).

### qRT-PCR analysis

Total RNA was extracted from different tissues using TRIzol regent (Invitrogen, Carlsbad, CA, United States) and first-strand cDNA were synthesized from 1.5 μg total RNA using the PrimeScript™ RT reagent kit with gDNA Eraser (TaKaRa, Dalian, China) according to the manufacturer’s instructions. All the primers used for the qRT-PCR analysis are shown in Supplementary Table 2. The PCR products were sequenced to verify the specificity of a cloned fragment of the target genes. The *sorghum eukaryotic translation initiation factor 4*α (*SbEif4*α) was used as the internal control. All reactions were performed *via* the Bio-Rad CFX96 real-time system, and the relative transcription levels were calculated *via* the method of 2^–Δ^ ^Δ^ *^CT^* based on the expression of *SbEif4*α (Livak and Schmittgen, 2001).

### Co-expression analysis

The co-expression profiling was performed according to Fu and Xue (2010). The Type I sorghum starch biosynthesis-related genes were considered the guide genes, and Pearson correlation coefficient (PCC) between the guide genes and regulators was calculated by Microsoft-Excel 365 based on the RNA-seq data. The threshold of PCC was set to 0.6, and Cytoscape 3.7.0 was used for the graphical results display.

### Promoter isolation and gene cloning

DNA from leaf samples of BTx623 was isolated by the cetyltrimethylammonium bromide (CTAB) method for promoter cloning. The mixed cDNA of different sorghum tissues was used as the templates for gene cloning. The promoters of starch biosynthesis-related genes and TFs were amplified *via* PCR reaction system of 20 μL with KOD DNA polymerase (Toyobo, Osaka, Japan). The PCR products were purified with Gel Extraction Kit D2500 (OMEGA, United States), and subsequently cloned into the pMD19-T vector (TaKaRa, Dalian, China) for sequencing. All primers of cloned genes are shown in Supplementary Table 2.

### Sub-cellular localization of candidate transcription factors

Vector of pCAMBIA2300-35S-eGFP was used for the sub-cellular localization analysis. The regulators were amplified with the primers (Supplementary Table 2) containing *Bam*HI and *Xba*I sites without the termination codon, and the PCR products were then subcloned into pCAMBIA2300-35S-eGFP to form the fusion protein of regulatory factor and enhanced green fluorescent protein (eGFP). All these vectors were constructed *via* ClonExpress^®^ MultiS One Step Cloning Kit (Vazyme, Nanjing, China).

The pCAMBIA2300-35S-CTF-eGFP was transformed into protoplast through the method of PEG-Ca^2+^ (Chen et al., 2016). The sub-cellular localization of eGFP and eGFP fusion proteins was performed under blue excitation light at 488 nm by a fluorescence microscope LSM 800 with Airyscan (Zeiss, Jena, Germany).

### Detection of TFBS motifs of sorghum starch biosynthesis-related gene

About 2,000 bp upstream the transcription start site (TSS) of sorghum starch biosynthesis-related genes was extracted from reference genome sequences through the Gramene database (See text footnote 1). The *cis*-acting regulatory elements (*cis*-elements) were predicted among the sequences of putative promoter regions using the PlantCARE (Lescot et al., 2002).

### Dual-luciferase assay in maize leaf protoplast

The pGreenII0800-LUC double-reporter vector and pBI221 were used to detect the relationship between promoter activity and regulatory factors. The promoter of *SbBt1* (1956 bp) and *SbGBSSI* (1902 bp) was subcloned into the pGreenII0800-LUC vector and drove *Luciferase* (*Luc*) expression. The full coding sequence of candidate TFs (CTF) was amplified and integrated into the pBI221 carrier and constructed the vector of TF expression driven by the *Ubi* promoter. The pUbi-CTF:pGreenII0800-Pro-Luc (1:2) was set as the experimental group, and pUbi-Gus: pGreenII0800-Pro-Luc (1:2) as the control group. All the constructs were transformed into the maize protoplast according to Xiao et al. (2021). LUC and Renilla (REN) luciferase activities were measured *via* the dual-luciferase assay kit (Promega, WI, United States) and analyzed *via* GloMax_2020 (Thermo Fisher Scientific, Waltham, MA, United States). LUC/REN ratio was calculated to measure the relationship between experimental and control groups. Four independent experiments were performed and each independent experiment consisted of three replicates. The difference with SbCTF on the promoter activity of starch biosynthesis-related genes was tested by *t*-test.

### Obtaining recombinant proteins *via Escherichia coli*

Prokaryotic expression vector of pET32a (YouBio)^6^ was used for the prokaryotic expression of sorghum NAC-type-His fusion protein. All primers with *Bam*HI and *Hin*dIII sites used to insert NAC-type TFs are shown in Supplementary Table 11. *E. coli* strain of BL21 was used as host cells for prokaryotic expression. When the OD600 value of the bacterial cultures reached 0.6, isopropyl β-D-1-thiogalactopyranoside (IPTG), at a final concentration of 0.5 mmol L^–1^, was added to induce the expression of fusion protein. Cells were broken ultrasonically under 120 W for 20 min after overnight incubation at 16°C in a shaker at 100 rpm. The Ni-Agarose His label Kit (CWBIO, Beijing, China)^7^ was used to purify the recombinant proteins according to the manufacturer’s instructions.

### Electrophoretic mobility shift assay

It was documented that the CACATG motif and [C/T]ACG sequence in the target gene promoter tended to provide opportunities for *Arabidopsis* NAC TF to bind and respond to stress induction (Olsen et al., 2005; Puranik et al., 2012; Lindemose et al., 2014), and the ACGCAA motif was a core binding site of NAC regulators for regulating starch biosynthesis in rice and maize (Zhang et al., 2019; Wang et al., 2020). A double-strand DNA fragment (5′-TCACGCAACACGCAACACGCAACACGCAA G-3′) containing four tandem CACGCAA repeats was used to test whether sorghum NAC-type protein bound to the motif. The 5′ end of the DNA fragment was labeled with biotin in the company through synthesis (Sangon Biotech, Shanghai, China). The binding reaction and detection were performed according to the instructions of the Chemiluminescent EMSA Kit (Beyotime Biotechnology, Shanghai, China).

## Results

### Transcriptome sequencing and expression dynamics of tissue-specific genes

**FIGURE 1:**
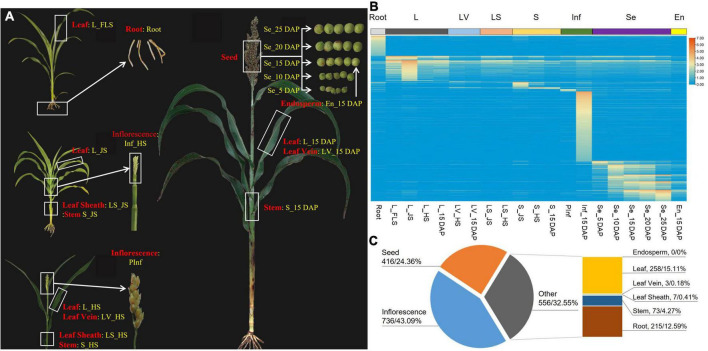
Tissue-specific expression patterns of detected genes *via* RNA-seq. **(A)** Tissues (red) and samples (yellow) for RNA-seq. **(B)** Heat map of the tissue-specific genes. **(C)** Categories of tissue-specific highly expressed genes (number of genes and the corresponding percentage (%) were separated with/).

To obtain the tissue-specific transcriptomic landscape of sorghum, eight tissues with a total of 20 samples of BTx623 were selected for RNA-sequencing (RNA-seq) (Figure 1A and Supplementary Table 3). For all tissues, high-quality RAN-seq datasets were obtained, and the unique map ratio ranged from 84.97% (En_15DAP) to 96.01% (LS_HS) (Supplementary Tables 4, 5). Meanwhile, more than 90% of reads were matched on exons, while those on introns and intergenic regions were less than 5% (Supplementary Table 5).

A total of 37,031 genes containing transcripts were detected across all 20 samples, namely, 34,118 (92.13%) original transcripts, 1,464 (3.95%) newly identified genes, and 1,449 (3.91%) small nuclear RNAs and small cytoplasmic RNAs (Supplementary Table 6). Among these genes, 17211 (46.48%) were found in all samples (Supplementary Table 6), and 1708 highly expressed genes exhibited tissue-specific patterns, namely, 736 highly expressed in inflorescence at the heading stage, 215 in the root, 208 in the leaf of jointing stage, 120 and 104 in corresponding whole grains of 25 DAP (days after pollination) and 10 DAP, and the number of specifically expressed genes in other tissues was less than 100 (Supplementary Tables 7, 8 and Figures 1B,C). No tissue-specific highly expressed genes were detected in the sample of En_15 DAP that belonged to endosperm (Figure 1C and Supplementary Table 8).

### Characterization of starch biosynthesis-related genes/enzymes in sorghum

**FIGURE 2:**
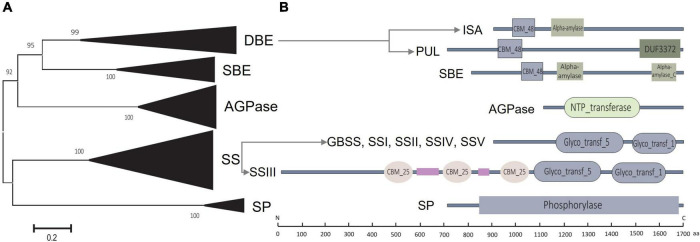
Phylogenetic and conserved domain analysis of starch biosynthesis-related enzymes. **(A)** Phylogenetic analysis of enzymes encoded by identified starch biosynthesis-related genes in sorghum. **(B)** Conserved domains are contained by the coding enzymes.

Twenty-seven sorghum starch biosynthesis-related genes were identified across the sorghum genome, namely, six for ADP-glucose pyrophosphorylase (AGPase), 10 for starch synthase (SS), four for both starch-branching enzyme (SBE) and starch-debranching enzyme (DBE), two for starch phosphorylase (SP), and one for Brittle-1 (BT1) protein (Supplementary Table 9). These genes are located across the entire genome of sorghum, and phylogenetic analysis showed that starch biosynthesis-related genes for corresponding enzymes tended to cluster in the same clade (Figure 2A and Supplementary Figure 1). Besides, all 27 starch biosynthesis-related genes in sorghum exhibited colinear trends (Supplementary Figure 1). Most of the colinear pairs were observed between sorghum and maize, while very few were detected within the sorghum genome or between sorghum and *Arabidopsis* (Supplementary Figure 1).

All the coding enzymes possessed peptide-chain lengths of 435 aa to 1,684 aa, with the molecular weight ranging from 46.83 kD to 190.02 kD, and pI varied from 5.01 to 9.33 (Supplementary Table 9). Conserved domain analysis revealed that both AGPase and SP possessed only one conserved domain named NTP transferase and phosphorylase at the amino acid sequence, separately, while the other enzymes contained two or more (Figure 2B). For example, all SSs contained two conserved domains of Glyco_transf_1 and Glyco_transf_5 that are located at the middle and C-terminal, while SSIII contained three additional unique CBM_25 domains at the middle peptide chain (Figure 2B). CBM_48 was a shared domain at the N-terminal of SBE and DBE (i.e., ISA and PUL), while both SBE and ISA contained conserved alpha-amylase domain, whereas domains of DUF3372 and alpha-amylase_C were detected at the C-terminal of PUL and SBE, respectively (Figure 2B).

Online dissection *via* MEME revealed that a total of 10 motifs (motifs 1 to 10) were possessed by the starch biosynthesis enzymes of sorghum (Sb), *Arabidopsis* (At), and maize (Zm). Each ortholog of AGPase and SP from Sb, At, and Zm presented all the 10 motifs that formed the corresponding conserved domain of NTP_transferase and Phosphorylase, while the other orthologs of these three species covered five to 10 motifs (Supplementary Figure 2, Figure 2B, Supplementary Table 10). The shared motifs of these enzymes among Sb, At, and Zm ranged from six to 10 except SSV which only two orthologs were detected between Sb and Zm with four shared motifs (Supplementary Figure 2). In addition, the shared motif number of Sb-At-Zm (among three species) almost kept the same as that of Sb-Zm (only between Sb and Zm), while the shared motifs of Sb-Zm exhibited more similar distribution features than those of Sb-At-Zm (Supplementary Figure 2).

### Dynamic expression patterns of identified genes

**FIGURE 3:**
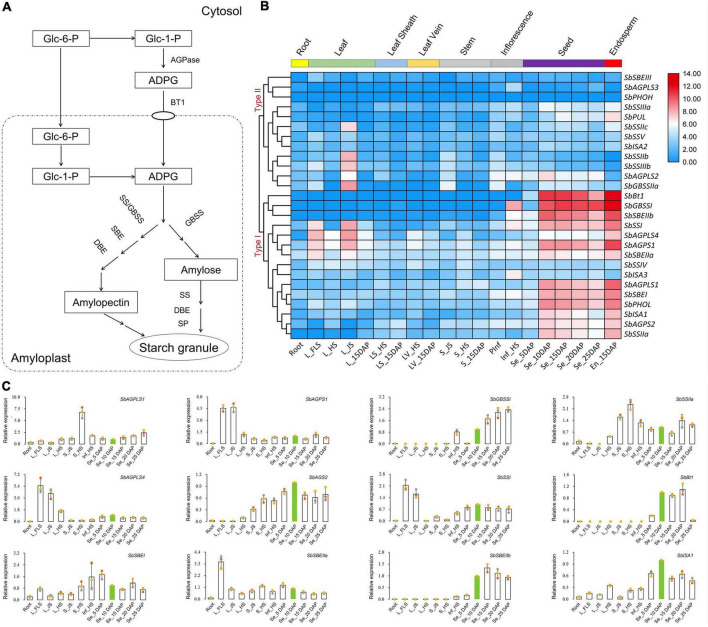
Heat map-based clustering analysis and expression dynamics dissection of 27 identified starch biosynthesis-related genes in sorghum. **(A)** Starch biosynthesis pathway in cereal endosperm (Jeon et al., 2010). **(B)** Heat map generated by hierarchical clustering *via* Pearson’s correlation as a measure of similarity. Red, white, and blue indicate high, medium, and low levels of gene expression, respectively. **(C)** qRT-PCR-based expression dynamics dissection of 12 selected genes highly expressed in grains *via* RNA-seq. Green-filled bars referred to the tissue/sample of Seed_10DAP with relative expression levels standardized to 1.

Starch biosynthesis pathway was well documented in the endosperm of cereal crops (Huang et al., 2021; Li et al., 2021; Figure 3A). The hierarchical clustering analysis indicated that 27 identified sorghum starch biosynthesis-related genes presented diverse expression levels among different tissues and development stages, and all these genes were divided into two categories according to their expression patterns (Figure 3B). Type I covered 15 genes that were almost all highly expressed in sorghum grains, while Type II contained the rest of 12 genes that exhibited relatively low expression levels in all the tissues (Figure 3B).

qRT-PCR was performed to reveal the expression features of sorghum starch biosynthesis-related genes, and the results showed that six genes of *SbAGPS2*, *SbBt1*, *SbGBSSI*, *SbSSIIa*, *SbISA1*, and *SbSBEIIb* were mainly expressed in seed samples of 5DAP to 25DAP (Figure 3C). Four genes of *SbAGPLS4*, *SbAGPS1*, *SbSSI*, and *SbSBEIIa* also presented transcripts during grain development, but their abundances were lower than those in leaves. Besides, *SbAGPLS1*, *SbSSIIa*, *SbISA3*, and *SbSBEI* exhibited various abundances of transcripts in all tissues under all detected development stages (Figure 3C).

### Screening and co-expression analysis of highly expressed transcription factors in sorghum grains

**FIGURE 4:**
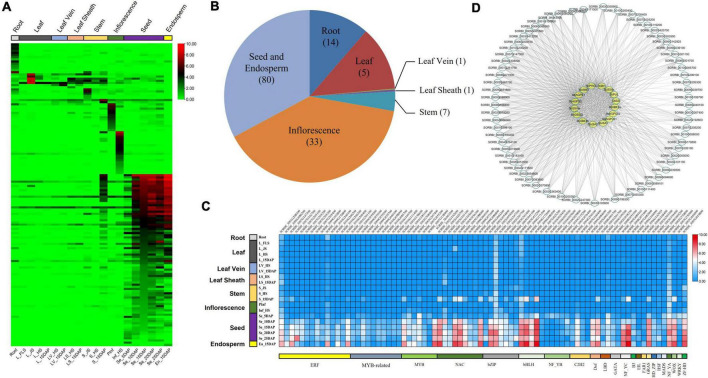
Tissue-specific detection of TFs in sorghum *via* RNA-seq. **(A)** Heat map showing the tissue-specific expression of TFs in sorghum. **(B)** Category of highly expressed TFs in sorghum. **(C)** Distribution of tissue-specific genes detected in all tissues and samples. Results of each tissue were summarized across multiple samples with corresponding development stages. **(D)** Co-expression analysis of TFs and sorghum starch biosynthesis-related genes.

Based on the results of RNA-seq, 141 highly expressed TFs were identified from different tissues (Figure 4A and Supplementary Table 11), namely, 14 from roots, seven from both leaves and stems, 33 from inflorescences, and 80 from grains and endosperm (Figure 4B). Eighty out of 141 identified TFs highly expressed in sorghum grains, belonged to 22 TF families, i.e., ERF (14), MYB (7) and MYB-related family (10), NAC (9), bHLH (6), bZIP and NF_YB (5), and C2H2 (4) (Figure 4C). The expression features of four TFs of NAC and eight TFs of other families were verified by qRT-PCR. The results showed that all the selected TFs exhibited relatively higher expression levels in sorghum grains and matched well with those observed *via* RNA-seq (Figure 4C and Supplementary Figure 3).

Co-expression analysis between 27 starch biosynthesis-related genes and grain highly expressed TFs indicated that 65 out of 80 TFs also co-expressed with 16 genes with correlation coefficients >0.6 (Figure 4D and Supplementary Table 12). Among these 16 genes co-expressed with 65 TFs, only one gene, i.e., *SbAGPLS2* belonged to Type II and was co-expressed with four TFs, while the remaining 15 genes belonged to Type I co-expressed with more than 40 TFs (Supplementary Table 12).

### Expression features and sub-cellular localization of NAC-type transcription factors

**FIGURE 5:**
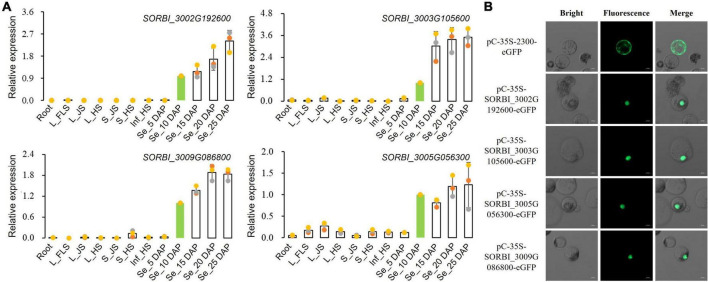
Expression analysis and sub-cellular localization of NAC TFs. **(A)** qRT-PCR assays of four NAC-type TFs among different tissues. **(B)** Sub-cellular localization of four selected NAC-type TFs in the protoplasts of maize leaves.

Four NAC TFs of SORBI_3002G192600, SORBI_3003G 105600, SORBI_3005G056300, and SORBI_3009G086800 with the highest expression levels *via* RNA-seq in sorghum grains were selected for further functional characterization. qRT-PCR results showed that all these four TFs presented higher expression levels in grains than other tissues, consistent with those detected through RNA-seq (Figure 5A and Supplementary Figure 4). Sub-cellular localization results of protoplast indicated that all these four TFs were located in the cell nucleus (Figure 5B).

### Globe *cis*-element detection and combine-verification of NAC transcription factors to *cis*-elements

*Cis*-element can provide a connection for signaling molecules and regulating gene transcriptions. The *cis*-element was scanned within the 2,000 bp upstream TSS of 27 sorghum starch biosynthesis-related genes to reveal the potential transcriptional regulation of these genes. The results showed that a total of 90 different types of *cis*-elements were detected at the target regions of 2,000 bp upstream TSS of 27 starch biosynthesis genes in sorghum (Supplementary Table 13). Nine out of these 90 *cis*-elements were captured by more than 20 genes, especially two *cis*-elements of G-box and MYB were captured by 26 genes (Supplementary Table 13).

**FIGURE 6:**
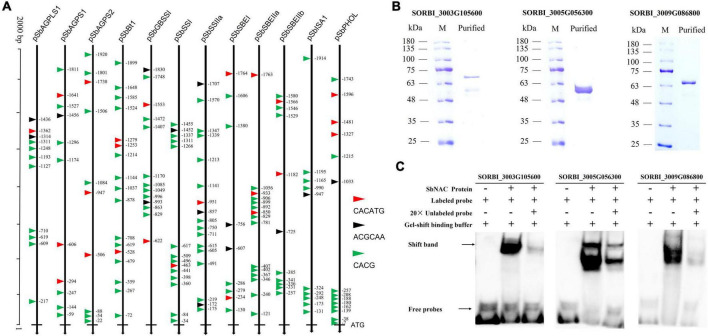
Binding site detection of NAC TFs and characterization of NAC TFs vs binding motifs. **(A)** The distribution of CACG core sequence, CACATG, and ACGCAA motif in promoter of sorghum starch biosynthesis-related genes; **(B)** NAC-type TF proteins obtained from prokaryotic expression system; **(C)** the binding results of NAC TFs with the ACGCAA motif repeats *in vitro*. “+” referred to the added composition and “–” to the corresponding component not added.

The CACATG motif and the motif containing the core sequence [T/C]ACG were identified as the core binding sites of stress-inducible NAC in *Arabidopsis* (Olsen et al., 2005; Puranik et al., 2012; Lindemose et al., 2014), while the ACGCAA motif was an important regulatory binding site of starch biosynthesis (Zhang et al., 2019; Wang et al., 2020). Twelve highly expressed genes in sorghum grains were selected for the scanning of the NAC-binding sites and the regulatory motif of ACGCAA within the 2,000 bp upstream TSS. The results showed that CACG and CACATG were widely detected within the 2000 bp upstream TSS of 12 genes, presenting uneven distribution trends (Figure 6A), while the ACGCAA motif was only captured by the target regions of nine out of 12 genes with the number of 1 (*SbAGPS1*, *SbSSI*, *SbSBEIIb*, *SbISA1*, and *SbPHOL*), 2 (*SbAGPLS1*, *SbGBSSI*, and *SbSBEI*), and 3 (*SbSSIIa*) (Figure 6A). Based on the prokaryotic system, we obtained the expressed proteins of three NAC TFs of SORBI_3003G105600, SORBI_3005G056300, and SORBI_3009G086800 (Figure 6B). The results of EMSA assay showed that all these three NAC TFs could directly bind to the CACGCAA motif *in vitro* (Figure 6C).

### NAC transcription factor activated the promoters of *SbBt1* and *SbGBSSI*

**FIGURE 7:**
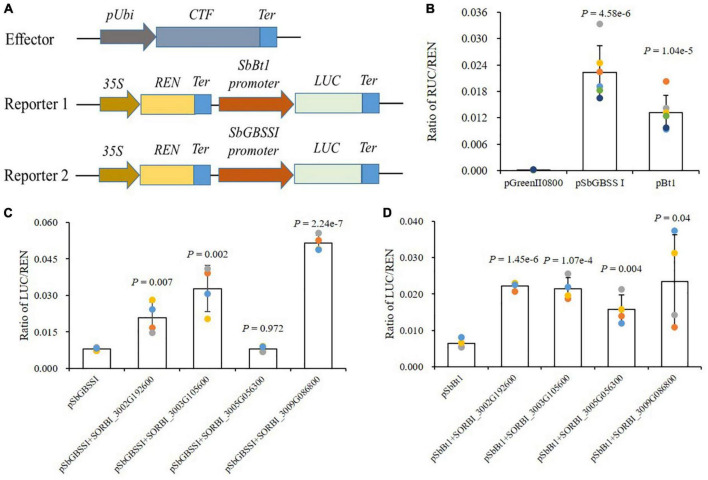
NAC-type TFs directly stimulate the promoter activities of *SbBt1* and *SbGBSSI*. **(A)** The schematic diagram of the effector and reporter vectors for dual-luc assays. pUbi, promoter of *Ubiquitin*; CTF, candidate transcription factor; LUC, Firefly luciferase; REN, Renilla luciferase; Ter, Terminator. **(B)** Detection of promoter activities of *SbBt1* and *SbGBSSI*. **(C,D)** Effect of NAC-type TFs on promoter activities of *SbBt1* **(C)** and *SbGBSSI* **(D)**, “+” means the co-transformation of the promoter (before +) and NAC TFs (after +). Dots indicate biological replications. Error bars indicate the S.D. of biological replications. *P*-values are determined by Student’s *t*-test.

BT1 and GBSSI are responsible for the transport of ADPG and the synthesis of sugar chain, both playing important roles in starch biosynthesis. A dual-luciferase (dual-luc) reporter assay was performed to detect whether the promoters of *SbBt1* and *SbGBSSI* could be directly activated by sorghum NAC-type transcription factor. After the confusion of promoters and guiding genes, the assays showed that promoters of *SbBt1* and *SbGBSSI* could drive the expression of *Luc* and improve the enzyme activity (Figures 7A,B). For *SbGBSSI*, the confusion with the three NAC TFs of SORBI_3002G192600, SORBI_3003G105600, and SORBI_3009G086800 significantly promoted its promoter activities (*P* < 0.01, Figure 7C), while the TF of SORBI_3005G056300 presented the non-significant promoting activity to the promoter of *SbGBSSI* (*P* = 0.972, Figure 7C). Differed from SbGBSSI, all four NAC TFs possessed significant or extremely significant promoting activities to the promoter of *SbBt1* (Figure 7D).

## Discussion

Starch is an important storage component in the grains of cereal crops and the main energy source for humans and animals. The biosynthetic pathway of starch in the endosperm is highly conserved in cereals (Huang et al., 2021). Functional enzymes, namely, AGPase, SS, SBE, DBE, and SP, are the core elements for the formation of substrates, the synthesis of polysaccharide chains, and the formation of starch granules (Zeeman et al., 2010; Bahaji et al., 2014; Toyosawa et al., 2016). The conservation of functional enzyme was not only reflected in the three major cereal crops of maize, rice, and wheat (Jeon et al., 2010; Huang et al., 2021) but also in barley (Radchuk et al., 2009), potato (Harsselaar et al., 2017), and other crops (Saithong et al., 2013). Compared with maize and rice, the functional enzymes of the starch biosynthesis pathway in sorghum are also conserved (Campbell et al., 2016). In this study, we identified a total of 27 genes encoding the key enzymes of starch biosynthesis from ADPG synthesis to amylopectin formation in sorghum (Supplementary Table 9), which agreed well with the results documented by Campbell et al. (2016). Meanwhile, the sequence characteristic analysis also further supports the conservation of the starch pathway in sorghum (Figure 2 and Supplementary Figure 3). The conservation of the starch biosynthesis pathway is also manifested in the expression pattern of starch biosynthesis-related genes. In rice, starch biosynthesis-related genes can be divided into Type I (in seeds) and Type II (in vegetative tissues; source tissues) based on gene expression patterns (Fu and Xue, 2010), and the same trends were also observed in maize (Xiao et al., 2021). The gene expression patterns related to starch biosynthesis detected in sorghum exhibited similar features to those in rice and maize, which can be grouped into Type I (highly expression in grains) and Type II (all tissues with certain expression levels) (Figure 3B). Widely co-expression networks were detected between most of these highly expressed genes and TFs, especially those from Type I, which provided informative guidelines for the further dissections of the molecular mechanism of starch biosynthesis-related genes at transcriptional levels (Hirai et al., 2007; Fu and Xue, 2010).

Starch biosynthesis is a conserved physiological process that depends on a series of functional enzymes. However, these functional enzymes are widely regulated by the allosteric regulation of metabolites, redox regulation, protein interaction, and reversible protein phosphorylation (Kötting et al., 2010). Meanwhile, the regulation of related genes at the transcriptional level becomes very important for starch biosynthesis, namely, the identification of *cis*-elements, the discovery of transcriptional regulatory factors, and the dissection of regulatory mechanism (Huang et al., 2021). Schmidt and colleagues reported that the rice TF of SERF1 (SALT-RESPONSIVE ERF1) could bind directly to the upstream region of *GBSSI*, negatively controlling the expression of *GBSSI* and other starch biosynthesis-related genes, i.e., *SSI*/*IIIa* and *AGPL2*, resulting in decreased starch accumulation and smaller grains in rice (Schmidt et al., 2014). In *Arabidopsis*, two circadian-related TFs of AtCCA1 and AtLHY can bind to the promoter of *GBSSI* and control the expression of this gene (Tenorio et al., 2003; López-González et al., 2019).

GBSSI can catalyze ADP-glucose (ADP-Glc) to form starch panicles in both monocot and dicot plants, while another protein, i.e., BT1 then functions as the transporter of ADP-Glc into amyloplast for the catalyzing of GBSSI (Shannon et al., 1996; Kirchberger et al., 2008; Bahaji et al., 2019; Wang et al., 2019). In maize, the promoter region of *BT1* was reported to possess the binding site of ZmMYB14, which exhibits transcriptional activity to *ZmBT1* (Xiao et al., 2017). Besides ZmMYB14, diverse binding sites with other MYB TFs were also discovered in the promoter regions of *BT1* in maize (Xiao et al., 2017). Additionally, Wang and colleagues detected various motifs within the promoter regions of *TaBT1* in wheat (Wang et al., 2019), and different *cis*-acting regulatory elements, including the binding sites with TFs belonging to the families of NAC, MYB, MYC, HD-ZIP III, were uncovered within the 2000 bp upstream TSS of *SbBT1* in sorghum in this study (Figure 7A and Supplementary Table 13), implying a general regulating pattern of *BT1* involved in starch biosynthesis in plants.

At present, the TFs involved in starch biosynthesis were mainly concentrated in TF families of bZIP, i.e., OsbZIP58/RISBZ1 (Wang et al., 2013), Opaque2 (Zhang et al., 2016), ZmbZIP91 (Chen et al., 2016), TubZIP28 (Song et al., 2020); NAC, i.e., OsNAC20/26 (Wang et al., 2020), ZmNAC126/128/130 (Zhang et al., 2019; Xiao et al., 2021), and TaNAC019 (Gao et al., 2021); AP2, i.e., RSR1 (Fu and Xue, 2010) and ZmEREB156 (Huang et al., 2016); MADS of OsMADS6/7 (Zhang et al., 2010); DOF of rice RPBF (Kawakatsu et al., 2009) and ZmDOF3/36 (Qi et al., 2016; Wu et al., 2019), and other families such as ZmMYB14 (Xiao et al., 2017), OsbHLH144, OsNF_Y (Bello et al., 2019), and so on. Most of these reported TFs presented more transcripts in grains, and their expression patterns were similar to those of starch biosynthesis-related genes highly expressed in grains (Wang et al., 2013, 2020; Zhang et al., 2019; Xiao et al., 2021). In this study, some TFs highly expressed in sorghum grains were detected, and most of them belonged to TF families of ERF, MYB, NAC, bZIP, and bHLH (Figure 4C), and these TFs were also widely co-expressed with starch biosynthesis-related genes in grains of sorghum (Figure 4D). These results are consistent with those documented in maize and other crops (Huang et al., 2021), which also provided the potential regulation of these TFs to starch biosynthesis in sorghum.

Additionally, in recent reports, NAC is undoubtedly an important and intensively reported family of TFs involved in the transcriptional regulation of starch biosynthesis. In rice, OsNAC20/26 could bind to the starch biosynthesis-related genes directly in regulating gene expression, and the starch content decreased significantly in *osnac20*/*26* double mutant (Wang et al., 2020). OsNAC127 and OsNAC129 tended to form heterodimers in response to heat stress to participate in rice grain filling (Ren et al., 2021). ZmNAC126 (Xiao et al., 2021) and ZmNAC128/130 (Zhang et al., 2019) were reported to regulate gene transcriptions by binding to the promoters of starch biosynthesis-related genes, affecting starch accumulation and contents in maize kernels. TaNAC019, an endosperm-specific TF, was found to regulate starch accumulation by directly activating the expression of relevant genes (Liu et al., 2020; Gao et al., 2021). In this study, we also collected the expression information of the reported NAC TFs, i.e., OsNAC20/26, ZmNAC126/128/130, and TaNAC019, and found that all these TFs were highly expressed in grains or kernels (Supplementary Figure 5C) and agreed well with the expression patterns of four NAC TFs cloned in sorghum grains in this study (Figure 6). Meanwhile, it is found that ZmNAC126 and four sorghum NAC genes are located on the same evolutionary branch, while the four sorghum NAC TFs reported in this paper are on the same branch with ZmNAC128/130 and OsNAC20/26 in phylogenetic analysis (Supplementary Figure 5B), which may explain the conservation of homologous NAC protein function among different species. ZmNAC128/130 and OsNAC20/26 are considered redundant in function (Zhang et al., 2019; Wang et al., 2020), suggesting that the four highly similar NAC proteins in sorghum might have redundancy in function. Moreover, *in vitro* assay also revealed that three of four NAC TFs could bind to CACGCAA, which is the binding site of OsNAC20/26 (Wang et al., 2020) and ZmNAC128/130 (Zhang et al., 2019), and four sorghum NAC TFs could promote the activity of promoters of *SbGBSSI* and *SbBt1* (Figure 7), strongly suggest potential regulations of NAC TFs to starch biosynthesis in sorghum. The four sorghum NAC proteins could recognize the same motif and affect the activity of the same promoter, which further indicated that there might be redundancy in their function of them.

Starch is one of the most important storage components and polysaccharide energy, and its biosynthesis pathway was conserved in the vascular plant, especially the conserved functional enzymes involved in this pathway (Jeon et al., 2010; Stitt and Zeeman, 2012; Huang et al., 2021). Besides, more and more documents suggested that transcriptional regulations served as a vital regulation mode in starch biosynthesis (Huang et al., 2021). In this study, we found that 10 cereal NAC TFs of maize, rice, wheat, and sorghum exhibited high conservations in gene expression and evolution, and could bind to the special sites of promoter regions of target genes and act on the transcriptional regulation of starch biosynthesis. These findings suggested that those reported transcriptional regulations of starch biosynthesis might also be conservative between the well-documented cereal crops, i.e., maize and rice, and those not intensively documented. Though the confirmation of this hypothesis needs further assays in more crops, it still provides potential and practical guidelines for the thorough dissection of transcriptional regulation of starch biosynthesis in both sorghum and other crops.

## Conclusion

We provided a global landscape of gene expression patterns in 20 sorghum tissues at different developmental stages and dissected the potential transcriptional regulation profiles of all starch biosynthesis-related genes in sorghum, as well. Sixty-five TFs that belonged to 22 TF families in sorghum were identified to co-express with 16 starch biosynthesis-related genes. Meanwhile, four NAC TFs highly expressed in endosperm and grains were cloned, and they were homologous of *ZmNAC128* and *ZmNAC130* and encoded nuclear-localized proteins. In the protoplast of maize leaves, NAC TFs exhibited activities to promote *SbBT1* and *SbGBSSI* and regulated their transcription. These results confirmed that transcriptional regulation is an important way of starch biosynthesis in sorghum grains and provided informative references for further regulation mechanism dissection of sorghum starch biosynthesis-related genes.

## Data availability statement

The datasets presented in this study can be found in online repositories. The name of the repository and accession number can be found below: National Center for Biotechnology Information (NCBI) BioProject, https://www.ncbi.nlm.nih.gov/bioproject/, PRJNA866867.

## Author contributions

QX, TH, WC, KM, TL, FX, QM, HD, and ML performed the experiments. QX, TH, and ZL drafted the manuscript. QX and TH analyzed the data. QX, XN, and ZL provided ideas, designed the research, and edited the manuscript. All authors contributed to the article and approved the submitted version.

## Footnotes

1
http://www.gramene.org/

2
https://www.ncbi.nlm.nih.gov/

3
http://smart.embl-heidelberg.de/

4
https://meme-suite.org/meme/

5
https://web.expasy.org/com putepi/

6
http://www.youbio.cn/

7
https://www.cwbiotech.com/
