# Supplementary figures and images for: Profiling of transcriptional regulators associated with starch biosynthesis in sorghum (Sorghum bicolor L.)
Source: Front Plant Sci. 2022 Aug 30;13:999747. doi: 10.3389/fpls.2022.999747 (PMC9468648; doi:10.3389/fpls.2022.999747)

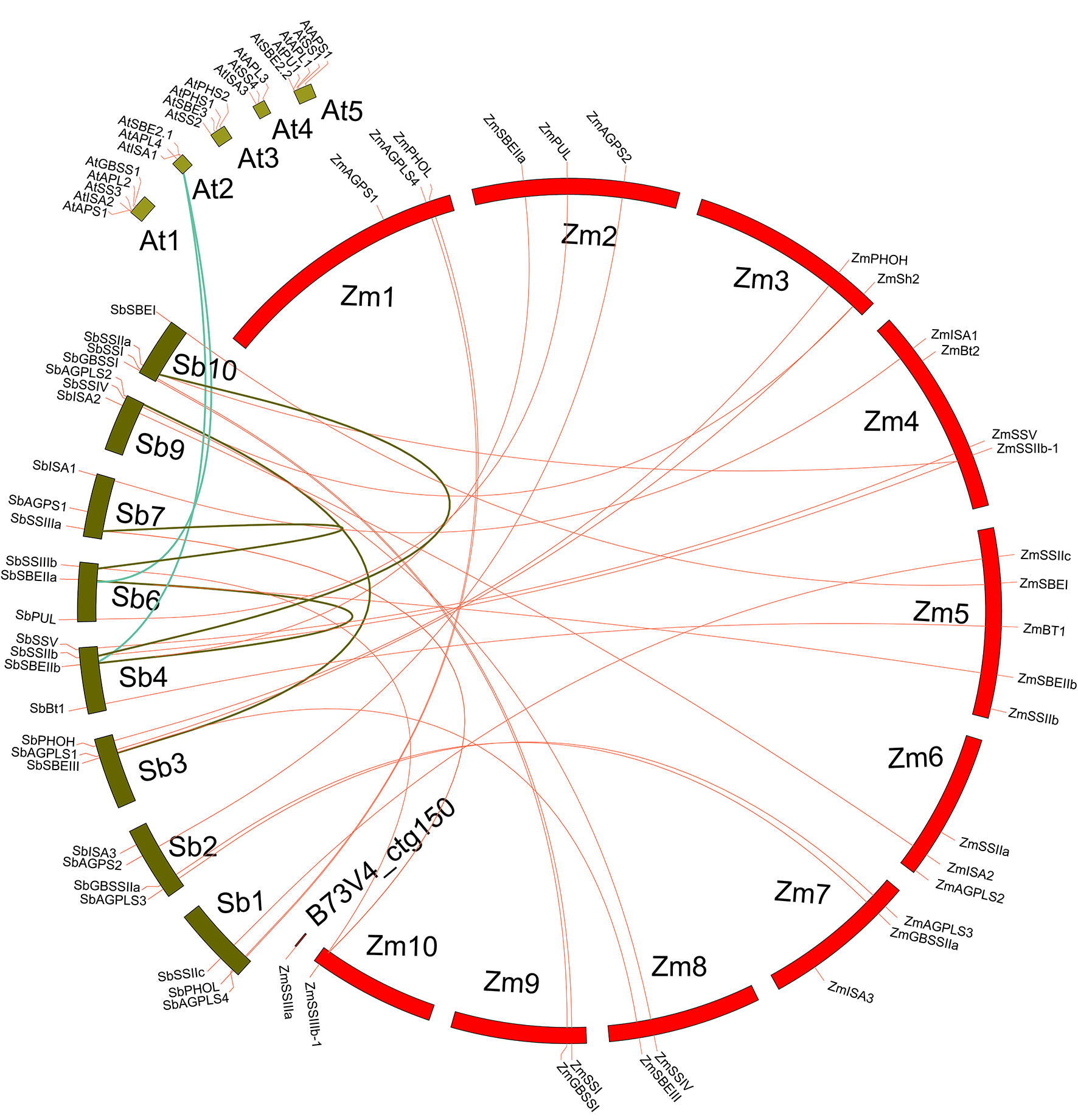

Supplement: Supplementary file 2 [file Image_1.TIF]

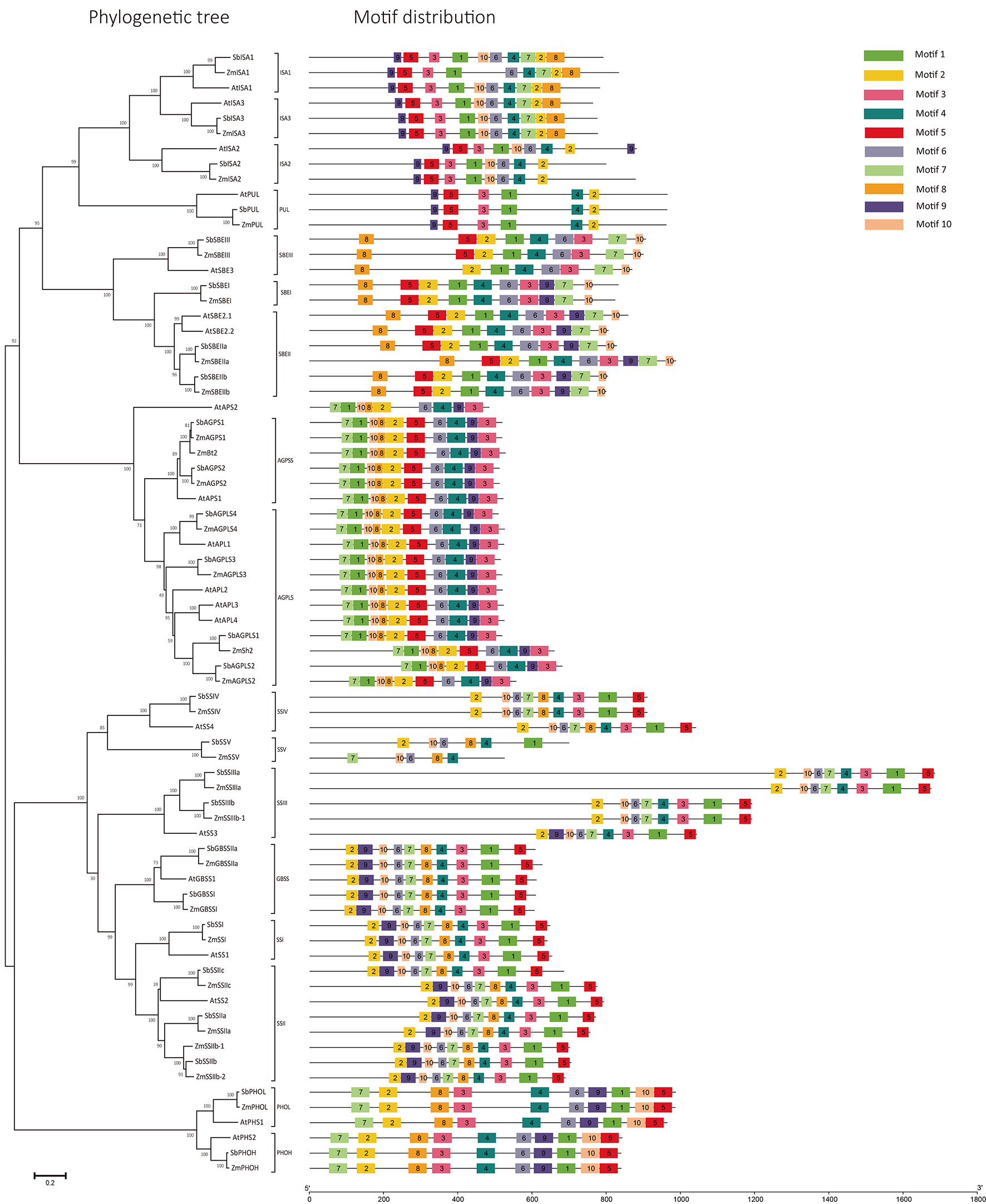

Supplement: Supplementary file 3 [file Image_2.TIF]

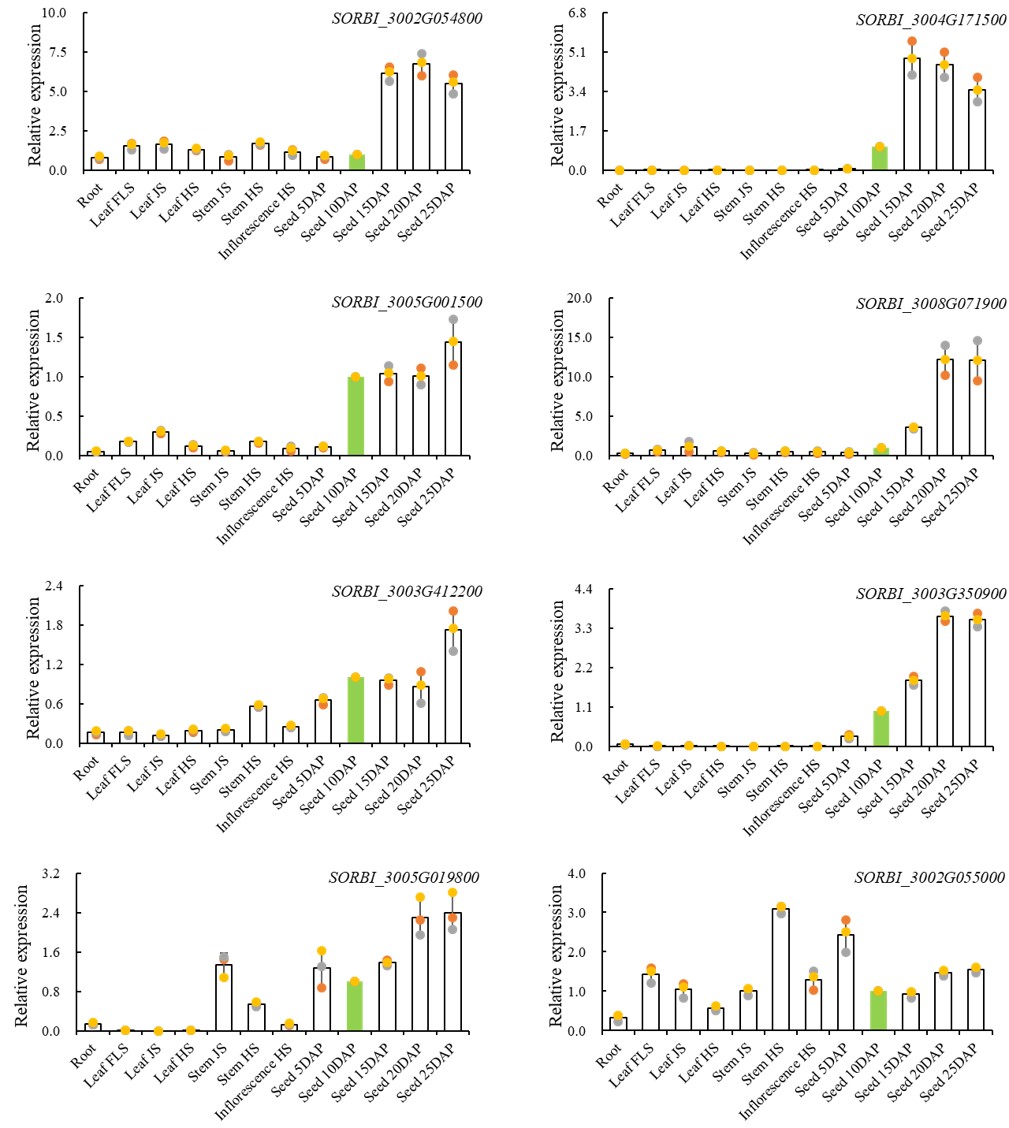

Supplement: Supplementary file 4 [file Image_3.JPEG]

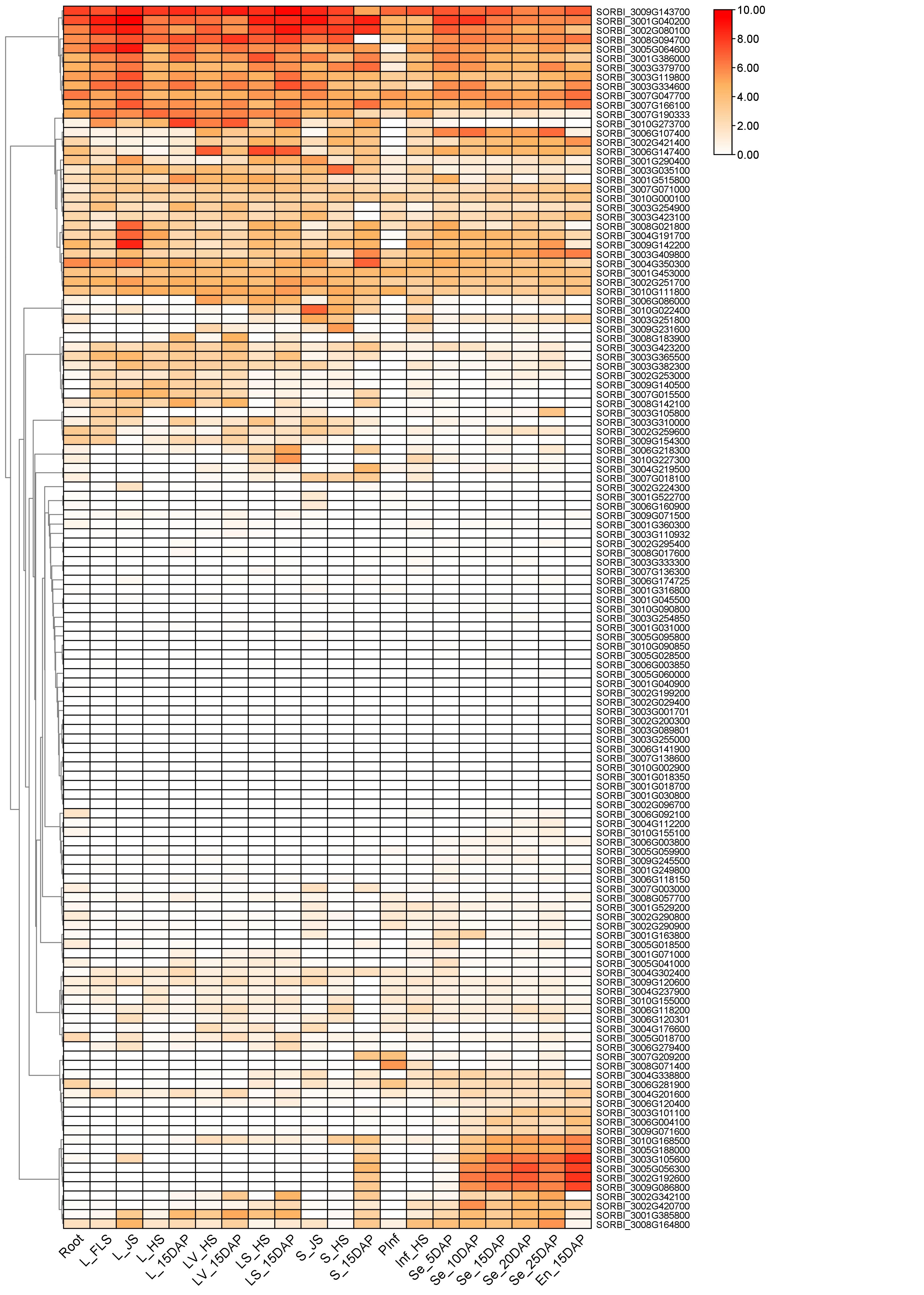

Supplement: Supplementary file 5 [file Image_4.JPEG]

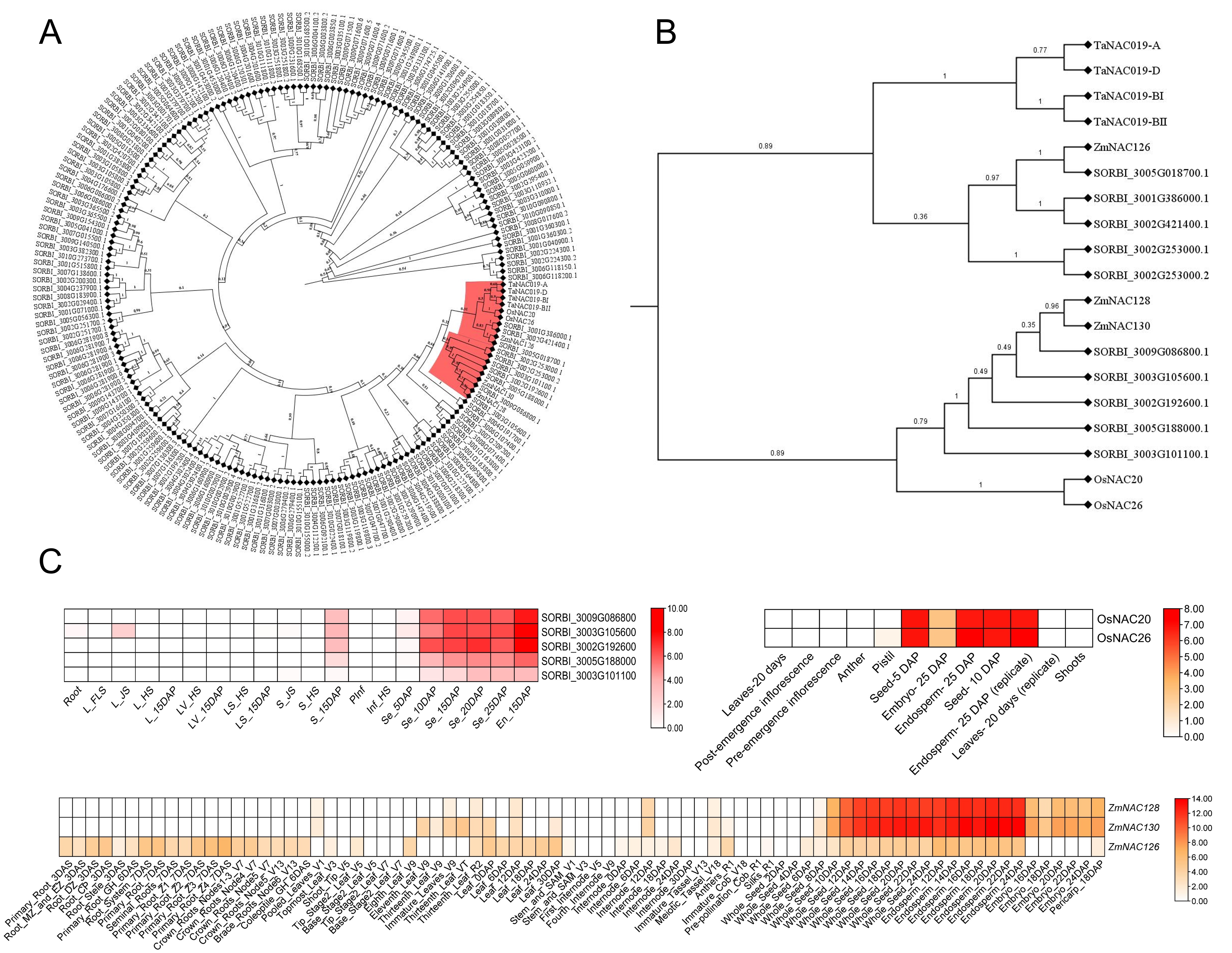

Supplement: Supplementary file 6 [file Image_5.JPEG]
